# Prognostic role of preoperative fluorine-18 fluorodeoxyglucose-positron emission tomography/computed tomography with an image-based harmonization technique: A multicenter retrospective study

**DOI:** 10.1016/j.xjon.2023.02.004

**Published:** 2023-02-14

**Authors:** Akira Hamada, Kazuhiro Kitajima, Kenichi Suda, Takamasa Koga, Junichi Soh, Hayato Kaida, Kimiteru Ito, Tetsuro Sekine, Kyoshiro Takegahara, Hiromitsu Daisaki, Masaki Hashimoto, Yukihiro Yoshida, Takanobu Kabasawa, Takashi Yamasaki, Seiichi Hirota, Jitsuo Usuda, Kazunari Ishii, Tetsuya Mitsudomi

**Affiliations:** a Division of Thoracic Surgery, Department of Surgery, Kindai University Faculty of Medicine, Osaka-Sayama, Japan; b Department of Radiology, Hyogo Medical University School of Medicine, Hyogo, Japan; c Department of Radiology, Kindai University Faculty of Medicine, Osaka-Sayama, Japan; d Department of Diagnostic Radiology, National Cancer Center Hospital, Tokyo, Japan; e Department of Radiology, Nippon Medical School, Musashi Kosugi Hospital, Kawasaki, Japan; f Department of Thoracic Surgery, Nippon Medical School Hospital, Tokyo, Japan; g Department of Radiological Technology, School of Radiological Technology, Gunma Prefectural College of Health Sciences, Maebashi, Japan; h Departments of Thoracic Surgery and Orthopedic Surgery, Hyogo Medical University School of Medicine, Hyogo, Japan; i Department of Thoracic Surgery, National Cancer Center Hospital, Tokyo, Japan; j Department of Pathological Diagnostics, Faculty of Medicine, Yamagata University, Yamagata, Japan; k Department of Surgical Pathology, Hyogo Medical University School of Medicine, Hyogo, Japan

**Keywords:** fluorine-18 fluorodeoxyglucose-positron emission tomography/computed tomography, harmonization, maximum standardized uptake value, non–small cell lung cancer, prognostic factor

## Abstract

**Objectives:**

Despite the prognostic impacts of preoperative fluorine-18 fluorodeoxyglucose-positron emission tomography/computed tomography examination, fluorine-18 fluorodeoxyglucose-positron emission tomography/computed tomography–based prognosis prediction has not been used clinically because of the disparity in data between institutions. By applying an image-based harmonized approach, we evaluated the prognostic roles of fluorine-18 fluorodeoxyglucose-positron emission tomography/computed tomography parameters in clinical stage I non–small cell lung cancer.

**Methods:**

We retrospectively examined 495 patients with clinical stage I non–small cell lung cancer who underwent fluorine-18 fluorodeoxyglucose-positron emission tomography/computed tomography examinations before pulmonary resection between 2013 and 2014 at 4 institutions. Three different harmonization techniques were applied, and an image-based harmonization, which showed the best-fit results, was used in the further analyses to evaluate the prognostic roles of fluorine-18 fluorodeoxyglucose-positron emission tomography/computed tomography parameters.

**Results:**

Cutoff values of image-based harmonized fluorine-18 fluorodeoxyglucose-positron emission tomography/computed tomography parameters, maximum standardized uptake, metabolic tumor volume, and total lesion glycolysis were determined using receiver operating characteristic curves that distinguish pathologic high invasiveness of tumors. Among these parameters, only the maximum standardized uptake was an independent prognostic factor in recurrence-free and overall survivals in univariate and multivariate analyses. High image-based maximum standardized uptake value was associated with squamous histology or lung adenocarcinomas with higher pathologic grades. In subgroup analyses defined by ground-glass opacity status and histology or by clinical stages, the prognostic impact of image-based maximum standardized uptake value was always the highest compared with other fluorine-18 fluorodeoxyglucose-positron emission tomography/computed tomography parameters.

**Conclusions:**

The image-based fluorine-18 fluorodeoxyglucose-positron emission tomography/computed tomography harmonization was the best fit, and the image-based maximum standardized uptake was the most important prognostic marker in all patients and in subgroups defined by ground-glass opacity status and histology in surgically resected clinical stage I non–small cell lung cancers.

## Graphical abstract

An image-based harmonization was the best fit compared with others, and SUVmax adjusted with image-based harmonization was the most important prognostic factor in all cohorts and in each subgroup. Perioperative treatment is a standard of care in patients with NSCLC with clinical stage II and III diseases. However, some patients with clinical stage I disease also show poor prognosis, and perioperative treatment may improve the outcomes of these patients. An image-based harmonization for FDG-PET/CT will facilitate universal application of FDG-PET/CT parameters to identify patients with poor prognoses. The 95% CIs are shown in shaded area. *FDG-PET/CT*, Fluorine-18 fluorodeoxyglucose-positron emission tomography/computed tomography; *SUVmax*, maximum standardized uptake value; *JSNM*, Japanese Society of Nuclear Medicine; *NSCLC*, non–small cell lung cancer; *MTV*, metabolic tumor volume; *TLG*, total lesion glycolysis; *CI*, confidence interval.

**Figure:**
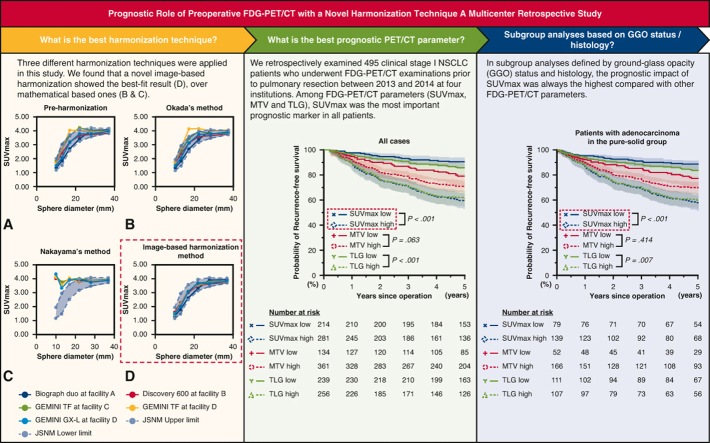

**Figure:**
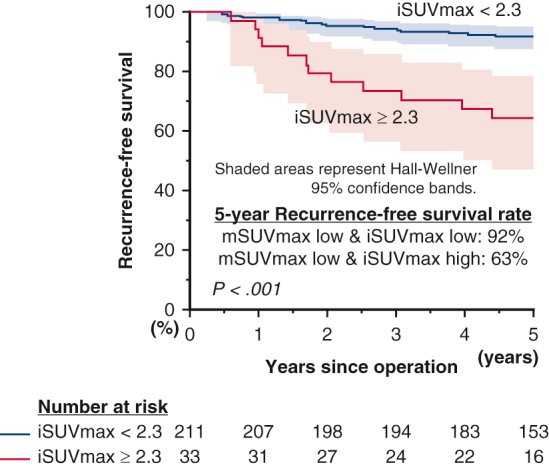
An image-based PET/CT harmonization appears superior to a conventional method.

**Central Message**

An image-based harmonization was the best fit compared with others, and SUVmax adjusted with image-based harmonization was superior to a conventional harmonization method in all cohort groups.

**Perspective**

Perioperative treatment is a standard of care in patients with NSCLC with clinical stage II and III diseases. However, some patients with clinical stage I disease also show poor prognosis and may be candidates for perioperative therapy. An image-based harmonization for FDG-PET/CT may identify these patients preoperatively, thereby facilitating the selection of patients for perioperative therapies.

Popularization of thin-section computed tomography (TS-CT) has accelerated the detection of small-sized lung cancers.^1^^,^^2^ Surgical resection is the standard of care for such early-stage non–small cell lung cancers (NSCLCs). However, there remains an appreciable risk of postsurgical recurrence even for patients with clinical stage I NSCLCs. Because these early-stage NSCLCs are heterogeneous regarding prognosis, numerous studies have tried to predict the prognosis of clinical stage I NSCLCs using parameters that can be obtained preoperatively.

Fluorine-18 fluorodeoxyglucose-positron emission tomography/computed tomography (FDG-PET/CT) is an almost mandatory clinical examination before pulmonary resection for patients with early-stage NSCLC to detect lymph node or distant metastases at a higher sensitivity and specificity compared with computed tomography alone or other radiological examinations.^3^, ^4^, ^5^ Additionally, FDG-PET/CT provides quantitative values that reflect the glucose uptake of the tumor, metabolic activity, and proliferative potential of cancer cells in the tumors. Using some of these quantitative values, such as maximum standardized uptake value (SUVmax), metabolic tumor volume (MTV), and total lesion glycolysis (TLG), many studies have reported that these parameters are significant prognostic markers in surgically resected patients with early-stage NSCLC.^6^, ^7^, ^8^, ^9^, ^10^, ^11^, ^12^ However, the quantitative values of these FDG-PET/CT parameters differ between institutions, which hampers clinical application of the FDG-PET/CT data as a tool to predict patients’ outcomes.

In early-stage NSCLCs, harmonization of FDG-PET/CT data has been attempted using mathematical-based harmonization methods^8^^,^^12^ that use an equation generated using an anthropomorphic body phantom that conformed to National Electrical Manufacturers Association standards^13^ to reduce inter- and intra-scanner variability. Nakayama and colleagues^8^ performed harmonization by adjusting the solid component sizes of the tumors because the deviation from the true standardized uptake value (SUV) depends on the solid component size of the tumors. In contrast, Okada and colleagues^12^ reported a harmonization method that calibrated SUVs by dividing the actual SUV by the SUVmean measured in the phantom background. However, these mathematical-based methods are considered inaccurate. Nakayama and colleagues’ method^8^ would be inadequate for smaller tumors, including those with ground-glass opacity (GGO) with a small solid component, and Okada and colleagues’ simplified method^12^ would be inadequate because the differences in SUVs between institutions are nonlinear. Therefore, image-based methods are becoming mainstream for FDG-PET/CT harmonization in other types of malignancies.^14^, ^15^, ^16^, ^17^, ^18^

In this study, we performed a multicenter retrospective study to evaluate the difference in SUVs between the 2 mathematical-based methods and an image-based harmonization method using phantom data. Because we confirmed that the image-based method was better than the mathematical-based methods, we performed further analyses to evaluate the validity and prognostic roles of the FDG-PET/CT data, which were harmonized using the image-based technique in surgically resected patients with NSCLC with clinical stage I disease. We also incorporated GGO status and a novel pathological classification, both of which have recently attracted attention as important prognostic factors for surgically resected NSCLCs.^1^^,^^2^^,^^19^^,^^20^

## Patients and Methods

### Inclusion Criteria

We retrospectively extracted patient information from clinical databases of Kindai University Hospital, Hyogo College of Medicine Hospital, National Cancer Center Hospital, and Nippon Medical School Hospital. Among patients who underwent surgical resection for clinical stage I NSCLC in accordance with the Union for International Cancer Control TNM Eighth edition guidelines^21^ between January 2013 and December 2014, we included 495 patients who underwent pretreatment FDG-PET/CT examinations before pulmonary resection. The exclusion criteria comprised any of the following: (1) received preoperative treatment; (2) confirmed small cell lung cancer; (3) underwent exploratory thoracotomy or biopsy alone; (4) positive surgical margins; and (5) history of lung cancer or synchronous lung cancer. We also excluded those who underwent FDG-PET/CT examination at another hospital.

The median follow-up period for all 495 patients was 67 months. The protocol of this study was reviewed by each Institutional Review Board (IRB), and approval was obtained at all 4 institutions: Kindai University Faculty of Medicine (IRB Number 31-172, November 26, 2019), Hyogo Medical University School of Medicine (IRB Number 3219, May 24, 2019). National Cancer Center Hospital (IRB Number 2019-082, August 17, 2019), and Nippon Medical School Hospital (IRB Number B-2019-065, November 8, 2019). The need to obtain written informed consent from the participants was waived because of the study's retrospective design.

### Thin-Section Computed Tomography Evaluation

For all patients, preoperative TS-CT images were independently reviewed by 2 investigators, and patients were classified into part-solid or solid groups based on the presence of a GGO component, as described in previous reports.^22^, ^23^, ^24^, ^25^, ^26^, ^27^ Computed tomography images were evaluated on a monitor with a window level of 600 to 700 Hounsfield units and a window width of 1500 to 2000 Hounsfield units. Solid components were defined as areas of increased opacification that completely obscured the underlying vascular structures on TS-CT images. GGO components were defined as areas of increased hazy density that did not obscure the underlying vascular structures.^28^^,^^29^

### Fluorine-18 Fluorodeoxyglucose-Positron Emission Tomography/Computed Tomography Examination and Harmonization Techniques

The participating institutions used different FDG-PET/CT scanner systems: Biograph Duo (Siemens Healthcare), Discovery 600 (GE Healthcare), Gemini TF (Philips Medical Systems), and Gemini GXL (Philips Medical Systems). Before the examination, patients fasted for at least 5 hours, and blood glucose was measured immediately before injection of FDG at 3.0 to 4.0 MBq/kg of body weight. None of the patients had a blood glucose level greater than 200 mg/dL. Approximately 60 minutes after the injection, static emission images were obtained, during which the patients were allowed to breathe normally. The experienced physicians (K.K. and H.K.), who were board certified in both diagnostic radiology and nuclear medicine and who were blinded to the other imaging results or clinical and histopathologic data, retrospectively reviewed all of the FDG-PET/CT images.

Regarding the techniques for FDG-PET/CT harmonization, 3 different harmonization methods were compared using mathematical-based^8^^,^^12^ and image-based methods. Image-based harmonization was performed using RAVAT (Nihon Medi-Physics Co, Ltd, Tokyo, Japan), which is a commercially available software package that harmonizes SUVs obtained with different PET/CT systems in a range advocated by the Japanese Society of Nuclear Medicine, using phantom data.^16^^,^^30^ Stand-alone RAVAT software can quantify PET images, and the software is typically used to adjust spatial resolution to harmonize PET images using a 3-dimensional Gaussian filter.

### Fluorine-18 Fluorodeoxyglucose-Positron Emission Tomography/Computed Tomography Parameters

**Figure E1:**
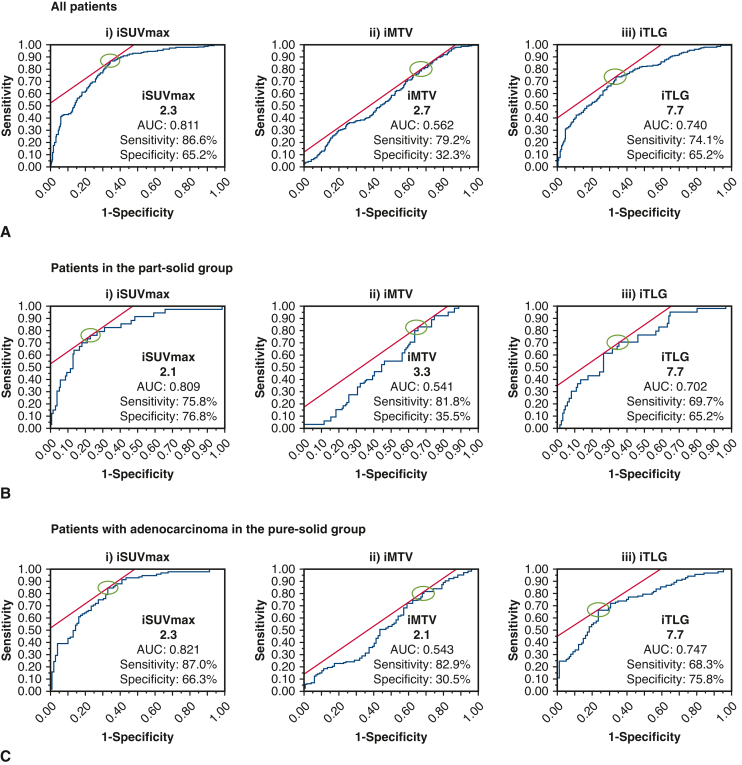

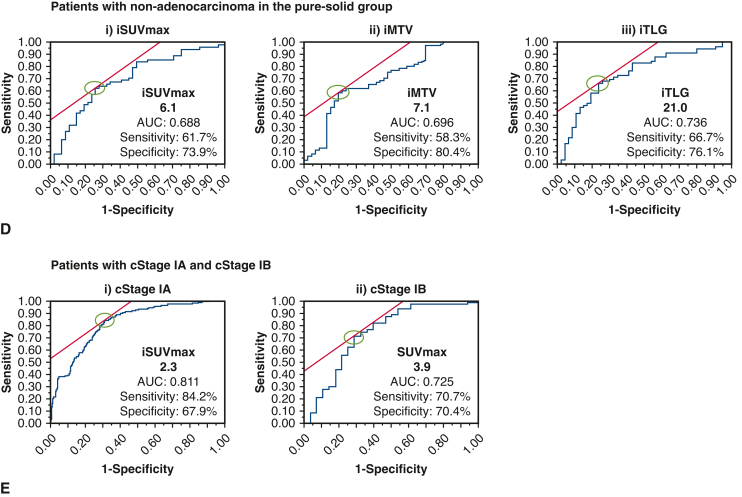

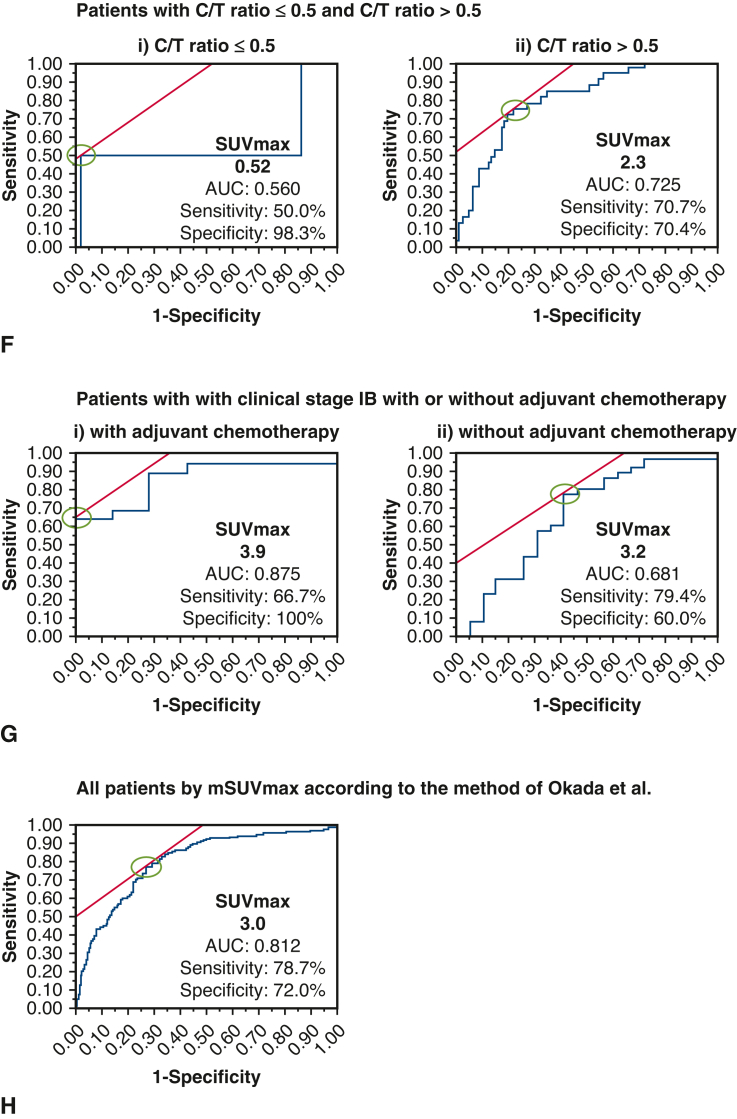
ROC curve for all patients (A), patients in the part-solid group (B), patients with adenocarcinoma in the pure-solid group (C), patients with nonadenocarcinoma in the pure-solid group (D), patients with clinical stage IA and stage IB (E), patients with C/T ratio 0.5 or less and C/T ratio greater than 0.5 (F), patients with clinical stage IB with or without adjuvant chemotherapy (G), and all patients by mSUVmax according to the method of Okada and colleagues^12^ (H). *iSUVmax*, Image-based maximum standardized uptake value; *AUC*, area under the curve; *iMTV*, image-based metabolic tumor volume; *iTLC*, image-based total lesion glycolysis; *cStage IA*, clinical stage IA; *cStage IB*, clinical stage IB; *C/T ratio*, consolidation/tumor ratio; *mSUVmax*, mathematical-based maximum standardized uptake value.

SUVmax was defined as maximum SUV within the target volume of the primary tumor and was determined using the following formula: concentration of radioactivity in the volume of interest (MBq/mL) × total body weight (kg)/injected radioactivity (g/MBq). The SUVmean was calculated as the summed SUV in each voxel in the target volume divided by the number of voxels within the target volume of the primary tumor. MTV was measured automatically inside the primary tumor volume of interest, with the margin threshold set at 40% of SUVmax. TLG was calculated as SUVmean × MTV, considering both metabolic activity and tumor burden. Image-based SUVmax (iSUVmax), image-based iMTV (iMTV), and image-based TLG (iTLG) were defined as the SUVmax, MTV, and TLG values calculated using the image-based harmonized FDG-PET/CT method in individual patients. Receiver operating characteristic (ROC) curves were used to identify optimal iSUVmax, iMTV, and iTLG cutoff values for predicting high pathologic invasiveness in all patients and in each subgroup (Figure E1).

### Pathologic Evaluation

Pathologic diagnoses were made by expert pathologists (T.Ka., T.Y., and S.H.) in accordance with the World Health Organization classification. Lung adenocarcinoma was classified as adenocarcinoma in situ, minimally invasive adenocarcinoma, and invasive adenocarcinoma, which was further divided into lepidic predominant, acinar predominant, papillary predominant, micropapillary predominant, solid predominant, or invasive mucinous adenocarcinoma.^19^ As previously reported, the predominant pattern was defined as the pattern with the largest percentage throughout the tissue sample. Invasive adenocarcinomas were further classified into 3 groups: grade 1, lepidic predominant; grade 2, acinar or papillary predominant; and grade 3, solid or micropapillary predominant, in accordance with the predominant pattern-based grading system.^19^

### Statistical Analyses

Statistical analyses were performed using JMP software, version 15.0 (SAS Institute Inc). Only simple statistical analyses were performed in this study, and following the recent guidelines,^31^^,^^32^ these analyses were performed by well-educated and experienced researchers. Continuous variables were compared using the Mann–Whitney *U* test, whereas categorical variables were compared using the chi-square test. ROC curves for iSUVmax, iMTV, and iTLG to predict lymphatic/vascular invasion, pleural invasion, or nodal involvement (high pathologic invasiveness) were generated to determine the cutoff values that yielded optimal sensitivity and specificity in accordance with a previously reported method.^12^ Recurrence-free survival (RFS) was defined as the interval from the day of surgery to the first event (relapse or death from any cause). For patients who did not experience disease recurrence, RFS was censored at the last visit. Overall survival (OS) was defined as the interval from the day of surgery to death from any cause. OS was censored at the last visit. RFS and OS were analyzed using the Kaplan–Meier method, and statistical differences in RFS or OS between groups were compared using the log-rank test. Univariate and multivariate Cox proportional hazard regression analyses were performed to assess the prognostic impact of the clinical parameters on RFS and OS.

## Results

### Comparison of an Image-based Versus Mathematical-based Harmonization

**Figure 1:**
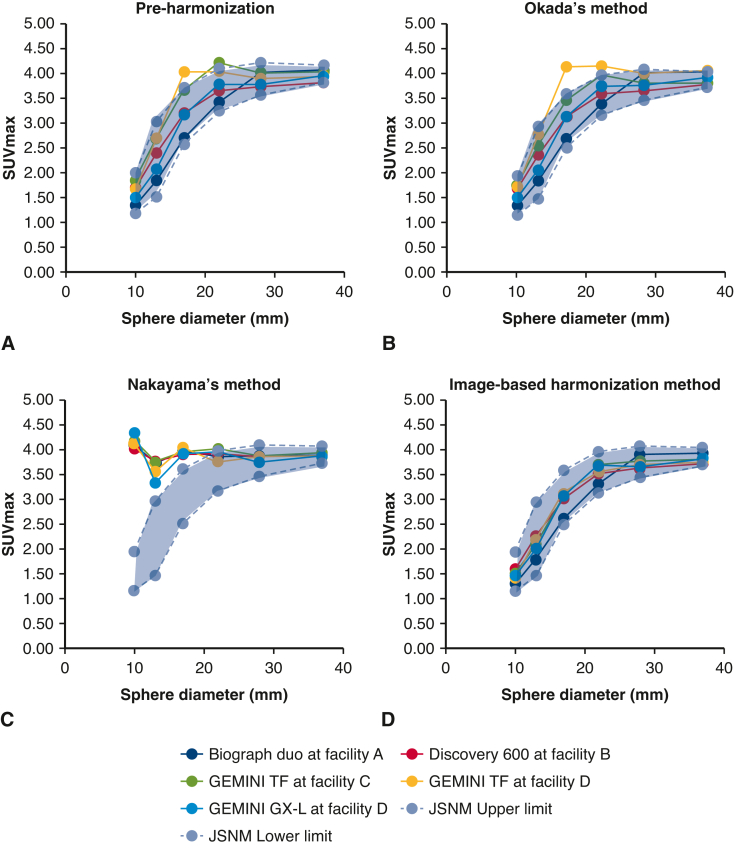
Recovery coefficient for 5 different FDG-PET/CT scanners obtained from 30-minute FDG-PET/CT imaging based on preharmonization (A), mathematical-based harmonization (B, Okada and colleagues’ method^12^; C, Nakayama and colleagues’ method^8^), and image-based harmonization (D). The *blue dashed lines* showed the Japanese Society of Nuclear Medicine reference range for a National Electrical Manufacturers Association body phantom. *SUVmax*, Maximum standardized uptake value; *FDG-PET/CT*, Fluorine-18 fluorodeoxyglucose-positron emission tomography/computed tomography; *JSNM*, Japanese Society of Nuclear Medicine.

We started our analysis by comparing the image-based harmonization approach with previously reported 2 mathematical-based approaches (ie, Okada and colleagues’ method^12^ and Nakayama and colleagues’ method^8^). We observed that harmonized SUVmax values for 5 different FDG-PET/CT scanners did not fall within the Japanese Society of Nuclear Medicine reference range for a National Electrical Manufacturers Association body phantom if harmonized using mathematical-based approaches (Figure 1, *A-C*). However, we found that the image-based approach provided the best-fit results (Figure 1, *D*). Therefore, we conclude that the image-based harmonization is preferable to mathematical-based harmonization, and we used the image-based method in the subsequent analyses.

### Patient Characteristics

The clinicopathological characteristics of the included patients are summarized in Table 1. Among the 495 patients, 79 (16%), 203 (41%), 128 (26%), and 85 (17%) had clinical stage IA1, IA2, IA3, and IB disease, respectively, in accordance with the current TNM classification (Eighth Edition). In our cohort, 324 patients (65%) had pure-solid tumors. Among the 495 patients, 421 (85%), 36 (7%), and 38 (8%) underwent lobectomy, segmentectomy, and wedge resection, respectively, and 383 patients (77%) underwent mediastinal lymph node dissection. Lymphatic invasion, vascular invasion, pleural invasion, and pathologic nodal involvement (pN1 or pN2) were recorded in 105 patients (21%), 172 patients (35%), 99 patients (20%), and 59 patients (12%), respectively.

**Table 1:** Characteristics of the 495 included clinical patients with stage I non–small cell lung cancer: clinicopathological findings by iSUVmax, iMTV, and iTLG values

Variables	Total (N = 495)	iSUVmax			iMTV			iTLG		
		<2.3 (n = 214)	≥2.3 (n = 281)		<2.7 cm^3^ (n = 134)	≥2.7 cm^3^ (n = 361)		<7.7 (n = 239)	≥7.7 (n = 256)	
Age (median), y	70	70	71	∗	70	70	ns	70	71	ns
Sex (male), n. (%)	268 (54)	100 (47)	168 (60)	∗∗	69 (51)	199 (55)	ns	117 (49)	151 (59)	∗
Smoking (yes), n. (%)	291 (59)	106 (50)	185 (66)	∗∗∗	71 (53)	220 (61)	ns	133 (56)	158 (62)	ns
Performance status (1), n. (%)	57 (12)	19 (9)	38 (14)	ns	15 (11)	42 (12)	ns	23 (10)	34 (13)	ns
GGO status (pure-solid), n. (%)	324 (65)	89 (42)	235 (84)	∗∗∗	93 (69)	231 (64)	ns	138 (58)	186 (73)	∗∗∗
Tumor size (>3 cm, ≤4 cm), n. (%)	85 (17)	18 (8)	67 (24)	∗∗∗	7 (5)	78 (22)	∗∗∗	11 (5)	74 (29)	∗∗∗
Histology (adenocarcinoma), n. (%)	389 (79)	204 (95)	185 (66)	∗∗∗	106 (79)	283 (78)	ns	212 (89)	177 (69)	∗∗∗
Operation (lobectomy)	421 (85)	169 (79)	252 (90)	∗∗	97 (72)	324 (90)	∗∗∗	186 (78)	235 (92)	∗∗∗
Nodal involvement (positive), n. (%)	59 (12)	10 (5)	49 (17)	∗∗∗	10 (7)	49 (14)	ns	10 (4)	49 (19)	∗∗∗
Lymphatic invasion (yes), n. (%)	105 (21)	14 (7)	91 (32)	∗∗∗	18 (13)	87 (24)	∗∗	27 (11)	78 (30)	∗∗∗
Vascular invasion (yes), n. (%)	172 (35)	15 (7)	157 (56)	∗∗∗	36 (27)	136 (38)	∗	43 (18)	129 (50)	∗∗∗
Pleural invasion (yes), n. (%)	99 (20)	15 (7)	84 (30)	∗∗∗	21 (16)	78 (22)	ns	28 (12)	71 (28)	∗∗∗
Pathological findings (high invasiveness), n. (%)	216 (44)	31 (14)	185 (66)	∗∗∗	45 (34)	171 (47)	∗∗	57 (24)	159 (62)	∗∗∗
Adjuvant chemotherapy (yes)	99 (20)	26 (12)	73 (26)	∗∗∗	14 (10)	85 (24)	∗∗	22 (9)	77 (30)	∗∗∗

*iSUVmax*, Maximum image-based standardized uptake value; *iMTV*, image-based metabolic tumor volume; *iTLG*, image-based total lesion glycolysis; *ns*, not significant; *GGO*, ground-glass opacity. ∗*P* < .05; ∗∗*P* < .01; ∗∗∗*P* < .001.

### Correlation Between Maximum Image-Based Standardized Uptake Value, Image-Based Metabolic Tumor Volume, and Image-Based Total Lesion Glycolysis and Clinicopathologic Findings

Cutoff values for iSUVmax, iMTV, and iTLG were determined using ROC curves that distinguish tumors with high pathologic invasiveness (Figure E1). Among 3 FDG-PET/CT parameters, iSUVmax had the highest sensitivity and specificity (area under the curve [AUC]: AUC = 0.811) in all patients compared with iMTV (AUC = 0.562) and iTLG (AUC = 0.740).

Next, we examined the correlations between these FDG-PET/CT parameters and clinicopathologic findings in all patients. A high iSUVmax was significantly correlated with male sex, smoking, pure-solid tumors, large tumor size, nonadenocarcinoma histology, pathologic lymph node metastasis, lymphatic invasion, vascular invasion, and pleural invasion (Table 1). The correlation between iMTV or iTLG and clinicopathologic characteristics is summarized in Table 1.

### Correlation Between Maximum Image-Based Standardized Uptake Value, Image-Based Metabolic Tumor Volume, and Image-Based Total Lesion Glycolysis Values and Pathologic Grading

We also analyzed the correlations between the FDG-PET/CT parameters and the histological findings. As shown in Table 2, most patients with squamous cell carcinoma (94%) were classified into the high iSUVmax group, whereas half of the patients with lung adenocarcinoma were classified into this group. Among lung adenocarcinomas, the percentages of patients who were classified into the high iSUVmax group (≥2.3) were 14%, 57%, and 83% for predominant pattern grade 1, grade 2, and grade 3 tumors, respectively. Such hierarchy was not evident in iMTV (66%, 79%, and 73%, for predominant pattern grade 1, grade 2, and grade 3, respectively) or in iTLG (28%, 53%, and 63%, respectively). These results suggest that a high iSUVmax value was the most important predictor of lung adenocarcinomas with higher pathologic grade.

**Table 2:** Characteristics of the 495 included clinical patients with stage I non–small cell lung cancer: details of pathologic subtypes by iSUVmax, iMTV, and iTLG values

	Total (N = 495)	iSUVmax		iMTV		iTLG	
Variables		<2.3 (n = 214)	≥2.3 (n = 281)	<2.7 cm^3^ (n = 134)	≥2.7 cm^3^ (n = 361)	<7.7 (n = 239)	≥7.7 (n = 256)
Squamous cell carcinoma, n. (%)	81	5 (6)	76 (94)	19 (23)	62 (77)	19 (23)	62 (77)
Adenocarcinoma, n. (%)	389	204 (52)	185 (48)	106 (27)	283 (73)	212 (54)	177 (46)
Adenocarcinoma subtypes, n. (%)							
Predominant pattern grade 1	93	80 (86)	13 (14)	32 (34)	61 (66)	67 (72)	26 (28)
AIS	13	12 (92)	1 (8)	9 (69)	4 (31)	11 (85)	2 (15)
MIA	25	25 (100)	0 (0)	13 (52)	12 (48)	22 (88)	3 (12)
Lepidic	55	43 (78)	12 (22)	10 (18)	45 (82)	34 (62)	21 (38)
Predominant pattern grade 2	214	92 (43)	122 (57)	46 (21)	168 (79)	101 (47)	113 (53)
Papillary	132	64 (48)	68 (52)	32 (24)	100 (76)	65 (49)	67 (51)
Acinar	82	28 (34)	54 (66)	14 (17)	68 (83)	36 (44)	46 (56)
Chi-square test for G1 vs G2		*P* < .001		*P* = .022		*P* < .001	
Predominant pattern grade 3	48	8 (17)	40 (83)	13 (27)	35 (73)	18 (37)	30 (63)
Micropapillary	12	3 (25)	9 (75)	4 (33)	8 (67)	6 (50)	6 (50)
Solid	36	5 (14)	31 (86)	9 (25)	27 (75)	12 (33)	24 (67)
Chi-square test for G2 vs G3		*P* < .001		*P* = .445		*P* = .263	
Variants	34	24 (71)	10 (29)	15 (44)	19 (56)	26 (76)	8 (24)
Others, n. (%)	25	5 (20)	20 (80)	9 (36)	16 (64)	8 (32)	17 (68)

Lepidic is invasive lepidic predominant adenocarcinoma. Papillary is invasive papillary predominant adenocarcinoma. Micropapillary is invasive micropapillary predominant adenocarcinoma. Solid is invasive solid predominant adenocarcinoma. *iSUVmax*, Maximum image-based standardized uptake value; *iMTV*, image-based metabolic tumor volume; *iTLG*, image-based total lesion glycolysis; *AIS*, adenocarcinoma in situ; *MIA*, minimally invasive adenocarcinoma.

### Correlation Between Maximum Image-Based Standardized Uptake Value, Image-Based Metabolic Tumor Volume, and Image-Based Total Lesion Glycolysis Values and Prognosis

**Figure 2:**
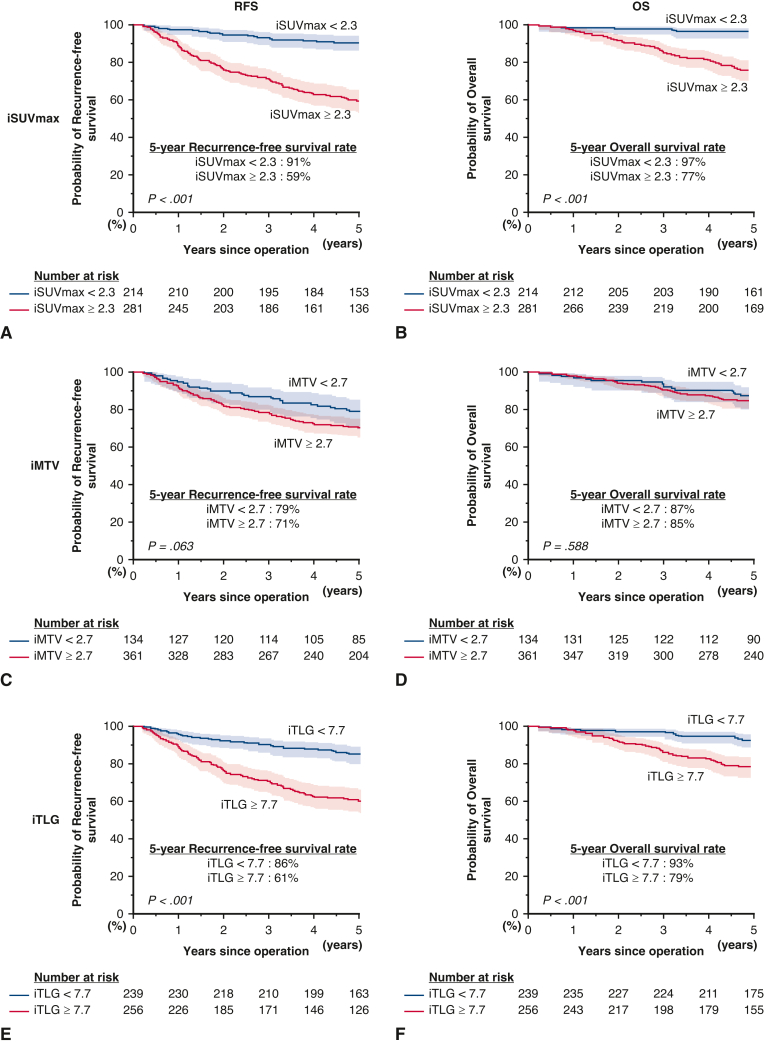
RFS and OS curves for all patients by iSUVmax (A and B), iMTV (C and D), and iTLG (E and F). The 95% CIs are shown in shaded area. *iSUVmax*, Image-based maximum standardized uptake value; *RFS*, recurrence-free survival; *iMTV*, image-based metabolic tumor volume; *iTLG*, image-based total lesion glycolysis; *OS*, overall survival; *CI*, confidence interval.

In the analyses of RFS and OS for the entire cohort, iSUVmax and iTLG values separated patients’ outcomes significantly (Figure 2). In the multivariate analysis, we found that iSUVmax (hazard ratio [HR], 3.02, *P* < .001 for RFS and HR, 3.66, *P* = .003 for OS), but not iTLG, was a significant prognostic factor (Table 3).

**Table 3:** Cox proportional hazard regression model results for recurrence-free survival and overall survival for all patients with clinical stage I non–small cell lung cancer

Variables	RFS				OS			
	Univariate		Multivariate		Univariate		Multivariate	
	HR (95% CI)	*P* value	HR (95% CI)	*P* value	HR (95% CI)	*P* value	HR (95% CI)	*P* value
Age (≥70 y vs <70 y)	1.42 (1.00-2.02)	.048	1.31 (0.92-1.88)	.140	1.86 (1.15-2.99)	.011	1.67 (1.02-2.73)	.041
Sex (male vs female)	1.76 (1.23-2.53)	.002	1.51 (0.95-2.39)	.079	2.87 (1.69-4.88)	<.001	2.45 (1.29-4.65)	.006
Smoking (yes vs no)	1.53 (1.06-2.21)	.022	0.97 (0.61-1.53)	.885	1.71 (1.05-2.81)	.032	0.85 (0.47-1.54)	.598
Performance status (1 vs 0)	1.48 (0.92-2.39)	.104			1.29 (0.66-2.51)	.458		
GGO status (pure-solid vs part-solid)	4.49 (2.66-7.57)	<.001	2.49 (1.41-4.39)	.002	5.72 (2.62-12.46)	<.001	2.75 (1.19-6.39)	.018
Tumor size (>3 cm, ≤4 cm vs ≤3 cm)	2.88 (1.99-4.19)	<.001	1.87 (1.25-2.80)	.002	2.59 (1.58-4.24)	<.001	1.72 (1.01-2.94)	.047
Histology (adenocarcinoma vs nonadenocarcinoma)	0.50 (0.35-0.72)	<.001	1.17 (0.78-1.75)	.461	0.33 (0.21-0.53)	<.001	0.87 (0.52-1.47)	.612
Operation (lobectomy vs sublobar)	1.48 (0.87-2.54)	.151			0.92 (0.49-1.70)	.918		
Adjuvant chemotherapy (yes vs no)	2.09 (1.45-3.02)	<.001	1.53 (1.04-2.26)	.033	1.88 (1.16-3.06)	.011	1.48 (0.88-2.48)	.142
iSUVmax (≥2.3 vs <2.3)	5.64 (3.47-9.18)	<.001	3.02 (1.67-5.46)	<.001	7.21 (3.46-15.02)	<.001	3.66 (1.55-8.65)	.003
iMTV (≥2.7 cm^3^ vs <2.7 cm^3^)	1.48 (0.98-2.25)	.065			1.16 (0.68-1.97)	.589		
iTLG (≥7.7 vs <7.7)	3.22 (2.18-4.76)	<.001	1.29 (0.80-2.07)	.300	3.07 (1.82-5.17)	<.001	1.09 (0.58-2.02)	.793

*RFS*, Recurrence-free survival; *OS*, overall survival; *HR*, hazard ratio; *CI*, confidence interval; *GGO*, ground-glass opacity; *iSUVmax*, image-based maximum standardized uptake value; *iMTV*, image-based metabolic tumor volume; *iTLG*, image-based total lesion glycolysis.

**Figure 3:**
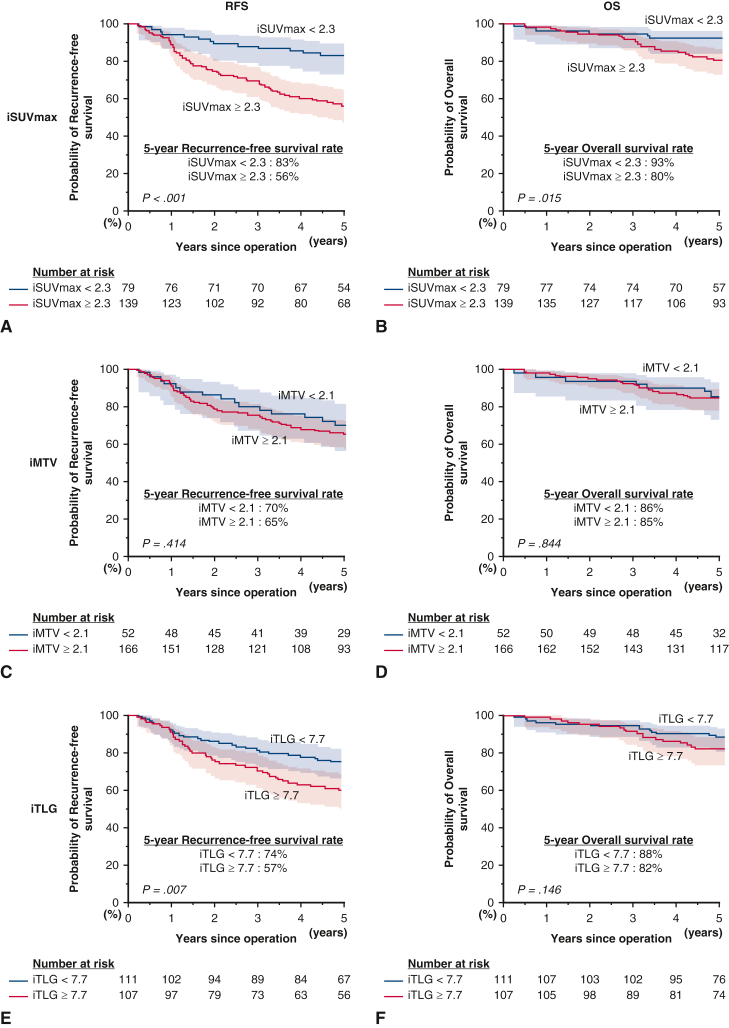
RFS and OS curves in patients with adenocarcinoma in the pure-solid group by iSUVmax (A and B), iMTV (C and D), and iTLG (E and F). The 95% CIs are shown in shaded area. *iSUVmax*, Image-based maximum standardized uptake value; *RFS*, recurrence-free survival; *iMTV*, image-based metabolic tumor volume; *iTLG*, image-based total lesion glycolysis; *OS*, overall survival; *CI*, confidence interval.

**Figure 4:**
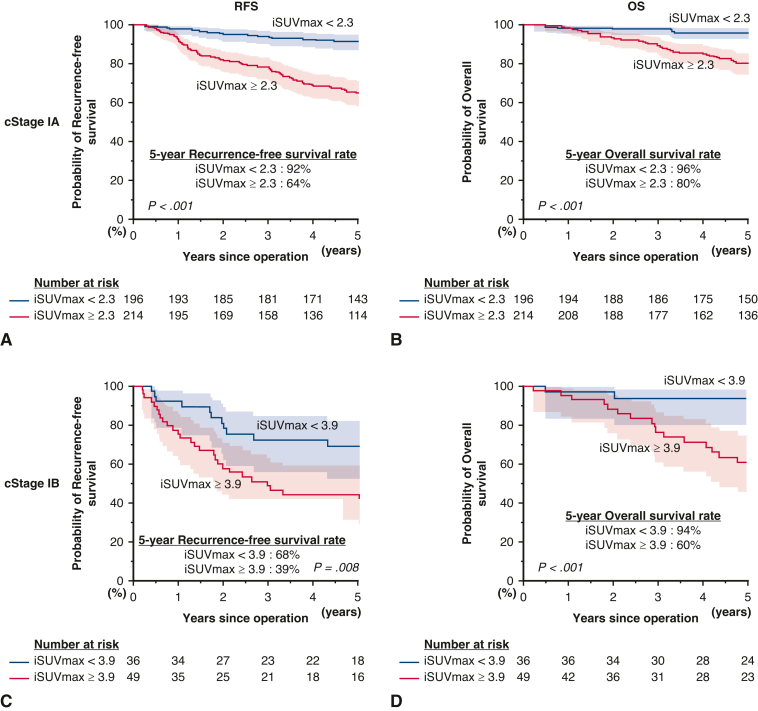
RFS and OS curves in patients with clinical stage IA (A and B) and IB (C and D) by iSUVmax. The 95% CIs are shown in shaded area. *cStage IA*, clinical stage IA; *RFS*, recurrence-free survival; *iSUVmax*, maximum standardized uptake value; *cStage IB*, clinical stage IB; *OS*, overall survival; *CI*, confidence interval.

**Figure E2:**
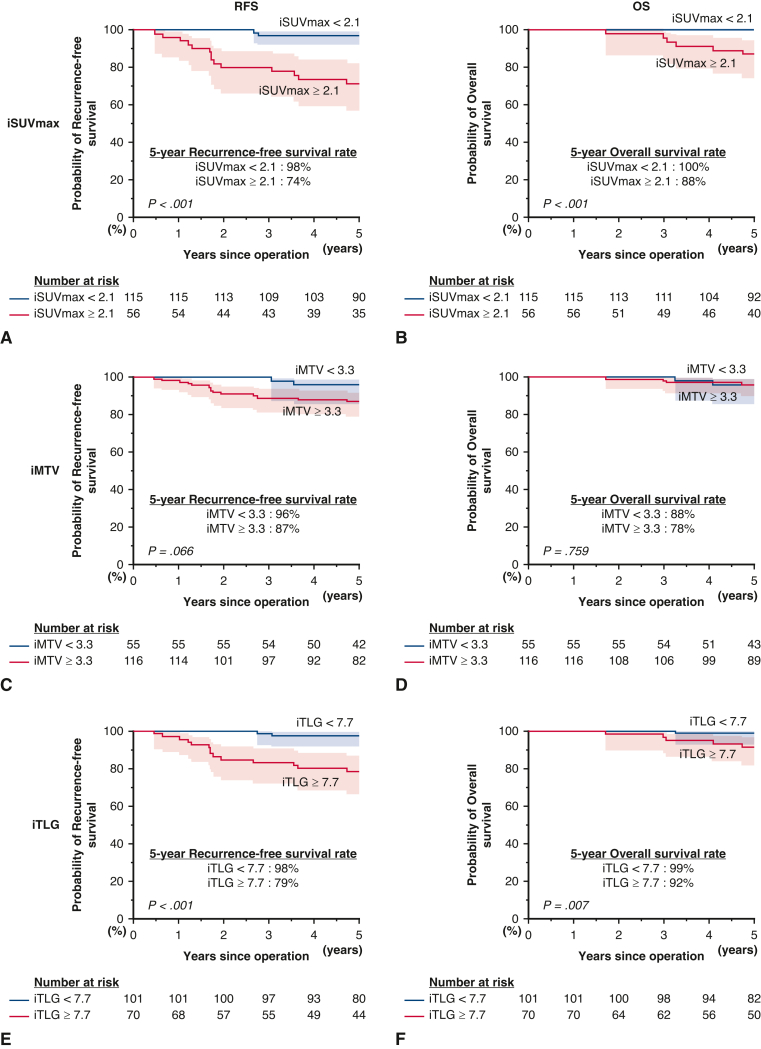
RFS and OS curves for patients in the part-solid group by iSUVmax (A and B), iMTV (C and D), and iTLG (E and F). The 95% CIs are shown in shaded area. *iSUVmax*, Image-based maximum standardized uptake value; *RFS*, recurrence-free survival; *OS*, overall survival; *iMTV*, image-based metabolic tumor volume; *iTLG*, image-based total lesion glycolysis; *CI*, confidence interval.

**Figure E3:**
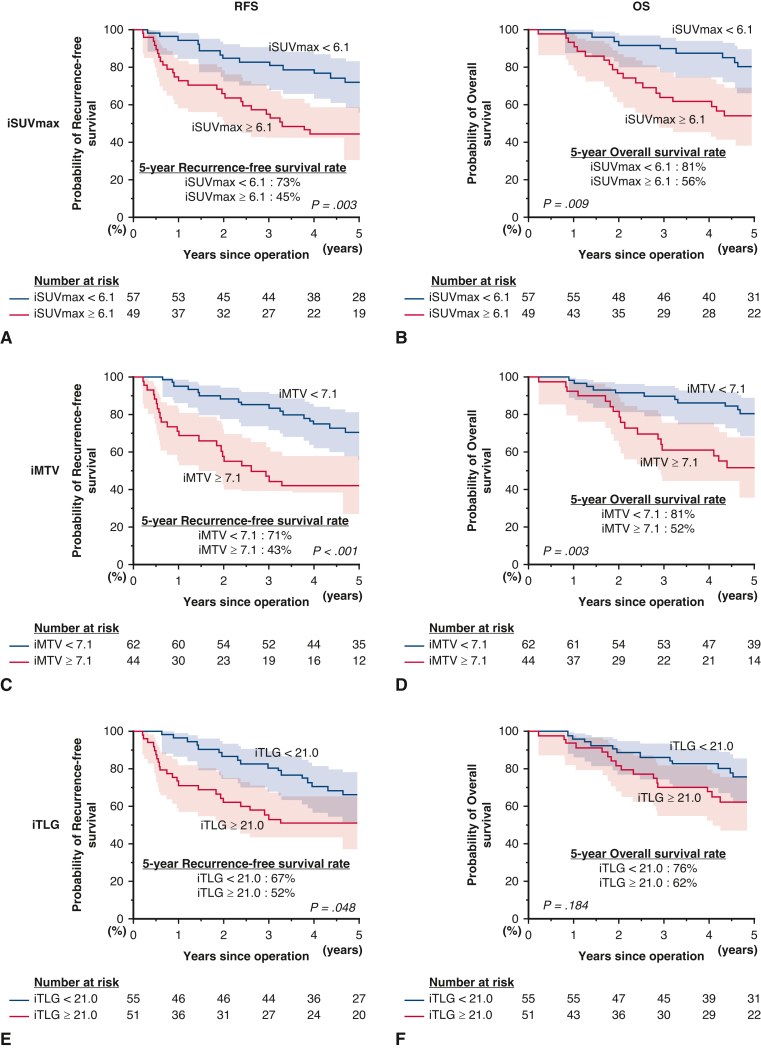
RFS and OS curves for patients with nonadenocarcinoma in the part-solid group by iSUVmax (A and B), iMTV (C and D), and iTLG (E and F). The 95% CIs are shown in shaded area. *iSUVmax*, Image-based maximum standardized uptake value; *RFS*, recurrence-free survival; *OS*, overall survival; *iMTV*, image-based metabolic tumor volume; *iTLG*, image-based total lesion glycolysis; *CI*, confidence interval.

**Figure E4:**
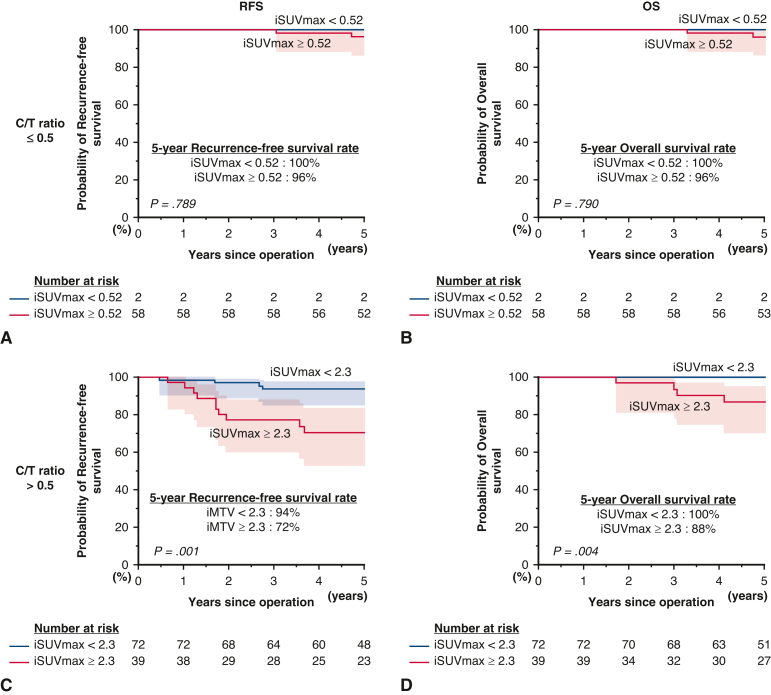
RFS and OS curves focusing on patients with part-solid group tumors by C/T ratio according to iSUVmax. A and B, RFS and OS for the patients with C/T ratio 0.5 or less. C and D, RFS and OS for the patients with C/T ratio greater than 0.5. ROC curve was also used to identify optimal iSUVmax cutoff values for predicting high pathologic invasiveness. The cutoff value for iSUVmax was set at 0.52 and 2.3 for the patients with C/T ratio 0.5 or less and with C/T ratio greater than 0.5, respectively. The 95% CIs are shown in shaded area. *C/T ratio*, Consolidation/tumor ratio; *RFS*, recurrence-free survival; *iSUVmax*, image-based maximum standardized uptake value; *OS*, overall survival.

**Figure E5:**
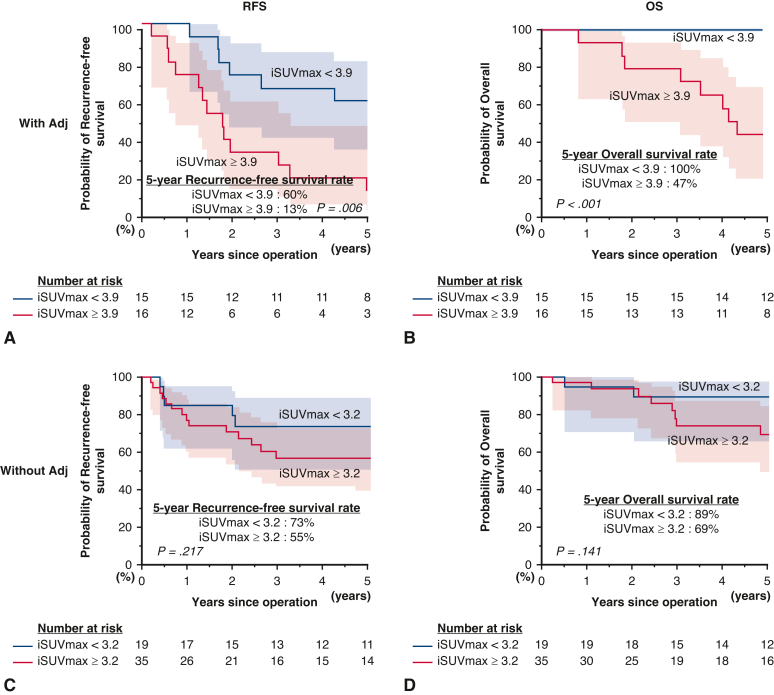
RFS and OS curves in patients with clinical stage IB with (A and B) or without (C and D) adjuvant chemotherapy by iSUVmax. ROC curves were also used to identify optional iSUVmax set at 3.9 and 3.2 for the patients with stage IB with adjuvant chemotherapy. The 95% CIs are shown in shaded area. *RFS*, Recurrence-free survival; *iSUVmax*, image-based maximum standardized uptake value; *OS*, overall survival.

**Figure E6:**
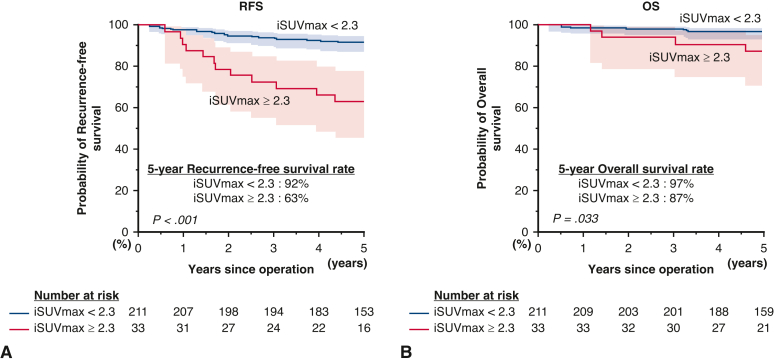
Subgroup analysis focusing on patients in low-risk group judged by a mathematical-based harmonization (Okada and colleagues’ method^12^). RFS (A) and OS (B) curves are shown based on the iSUVmax. The 95% CIs are shown in shaded area. *RFS*, Recurrence-free survival; *iSUVmax*, image-based maximum standardized uptake value; *OS*, overall survival; *CI*, confidence interval.

In the subgroup analysis focusing on pure-solid lung adenocarcinomas (Figure 3), a high iSUVmax value was again the only significant predictive factor of both poor RFS (*P* < .001) and OS (*P* = .015). In multivariate analysis, iSUVmax was a significant prognostic factor for RFS (HR, 3.18, *P* = .001) and OS (HR, 2.72, *P* = .017) (Table 4). In the other subgroups, such as the part-solid adenocarcinoma and nonadenocarcinoma groups, high iSUVmax was consistently a significant poor prognostic factor for both RFS and OS (Figures E2 and E3). In further subgroup analysis, we observed that iSUVmax was a significant prognostic factor in patients with part-solid adenocarcinoma (consolidation/tumor ratio [C/T ratio] >0.5), whereas those with C/T ratio 0.5 or less usually had low iSUVmax and had excellent survival outcomes (Figure E4). These results suggest that iSUVmax is an important prognostic factor to predict poor RFS and OS in patients with clinical stage I NSCLC and in subgroups defined by GGO status (excluding those with C/T ratio ≤ 0.5) or histology. In addition, iSUVmax was a significant prognostic factor in patients with clinical stage IA and IB NSCLC. It is of note that patients with clinical stage IB with high iSUVmax had the worst RFS (5-year RFS: high iSUVmax group, 39%; low iSUVmax group, 68%, *P* = .008; 5-year OS: high iSUVmax group, 60%; low iSUVmax group, 94%, *P* = .001) (Figure 4). Adjuvant chemotherapy, tegafur/uracil, or platinum doublet (if pathological nodal involvement was found) was administered for these patients according to the Japanese guideline and patients’ general conditions. We observed that high iSUVmax was associated with poor prognosis in patients with clinical stage IB disease irrespective of the administration of adjuvant chemotherapy (Figure E5). We also evaluated the ability of iSUVmax as a predictor of poor prognosis compared with a conventional mathematical-based SUVmax (mSUVmax).^12^ Among 244 patients in the low-risk group judged by the mSUVmax, 33 were reclassified into the high-risk group by iSUVmax. As shown in Figure E6, these patients showed poorer RFS and OS compared with patients who were low risk by both mSUVmax and iSUVmax, although there were only 3 patients who were low risk by iSUVmax but high risk by mSUVmax.

**Table 4:** Cox proportional hazards regression model for recurrence-free survival and overall survival in patients with adenocarcinoma in the pure-solid group

Variables	RFS				OS			
	Univariate		Multivariate		Univariate		Multivariate	
	HR (95% CI)	*P* value	HR (95% CI)	*P* value	HR (95% CI)	*P* value	HR (95% CI)	*P* value
Age (≥70 y vs <70 y)	1.12 (0.71-1.77)	.613			2.12 (1.10-4.10)	.026	2.16 (1.12-4.18)	.022
Sex (male vs female)	1.30 (0.82-2.06)	.260			1.84 (0.95-3.57)	.069		
Smoking (yes vs no)	1.06 (0.66-1.68)	.817			0.81 (0.43-1.53)	.517		
Performance status (1 vs 0)	1.17 (0.62-2.22)	.623			1.11 (0.43-2.85)	.829		
Tumor size (>3 cm, ≤4 cm vs ≤3 cm)	1.58 (0.91-2.74)	.108			1.38 (0.63-3.02)	.416		
Operation (lobectomy vs sublobar)	1.34 (0.64-2.78)	.439			0.75 (0.31-1.80)	.519		
Adjuvant chemotherapy (yes vs no)	1.71 (1.05-2.80)	.033	1.34 (0.79-2.26)	.274	1.58 (0.80-3.13)	.192		
iSUVmax (≥2.3 vs <2.3)	3.25 (1.79-5.92)	<.001	3.18 (1.58-6.41)	.001	2.67 (1.18-6.07)	.019	2.72 (1.20-6.18)	.017
iMTV (≥2.1 cm^3^ vs <2.1 cm^3^)	1.27 (0.72-2.23)	.415			1.08 (0.49-2.37)	.844		
iTLG (≥7.7 vs <7.7)	1.88 (1.18-3.01)	.008	0.93 (0.53-1.65)	.816	1.61 (0.84-3.10)	.150		

*RFS*, Recurrence-free survival; *OS*, overall survival; *HR*, hazard ratio; *CI*, confidence interval; *iSUVmax*, image-based maximum standardized uptake value; *iMTV*, image-based metabolic tumor volume; *iTLG*, image-based total lesion glycolysis.

## Discussion

**Figure 5:**
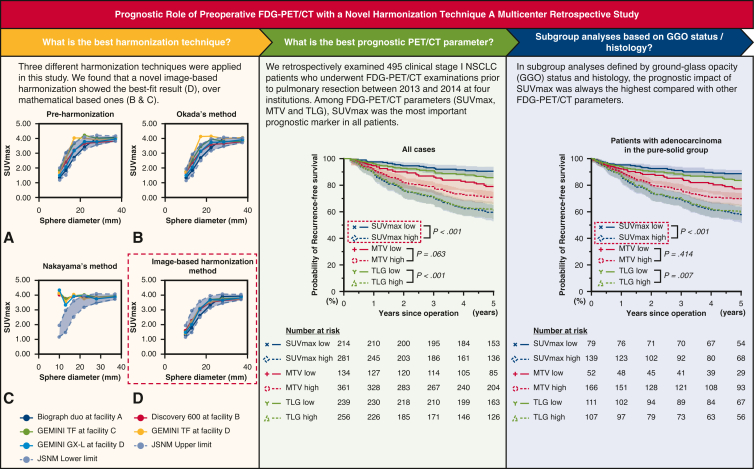
An image-based harmonization was the best fit compared with others, and SUVmax adjusted with image-based harmonization was the most important prognostic factor in all cohort and in each subgroup. Perioperative treatment is a standard of care in patients with NSCLC with clinical stage II and III diseases. However, some patients with clinical stage I disease also show poor prognosis and perioperative treatment may improve outcomes of these patients. An image-based harmonization for FDG-PET/CT will facilitate universal application of FDG-PET/CT parameters to identify these patients with poor prognosis. The 95% CIs are shown in shaded area. *FDG-PET/CT*, Fluorine-18 fluorodeoxyglucose-positron emission tomography/computed tomography; *SUVmax*, maximum standardized uptake value; *JSNM*, Japanese Society of Nuclear Medicine; *NSCLC*, non–small cell lung cancer; *MTV*, metabolic tumor volume; *TLG*, total lesion glycolysis; *CI*, confidence interval.

This clinical study uses an image-based harmonization method for FDG-PET/CT parameters to evaluate prognostic factors in patients who received pulmonary resection for clinical stage I NSCLCs in multi-institutions (with different FDG-PET/CT machines). We observed that the image-based harmonization method was superior to previously reported mathematical methods. In addition, we observed that iSUVmax was an important FDG-PET/CT parameter in terms of prognostic markers for RFS and OS as well as the predictor of histological grades among patients with lung adenocarcinoma. Last, we found that iSUVmax can identify patients with poor prognosis among those with low risk judged by a mathematical method (Figure 5).

The importance of SUVmax as a prognostic marker in clinical stage I diseases was reported in a previous study, although the main results of the study were the usefulness of MTV and TLG in the total cohort (clinical stage I-II NSCLCs) in multivariate analysis.^6^ Therefore, we consider that our result is consistent with the previous one, because our cohort enrolled only patients with stage I disease. In small-sized NSCLCs, it is hypothesized that the simple SUVmax, rather than the factors that include volume elements, would be more useful as a predictor of pathologic invasiveness, pathologic grade, and prognosis. Furthermore, it is of note that subgroup analysis in our study, based on histology and the GGO status, showed that iSUVmax was consistently better than iMTV and iTLG to predict RFS and OS. Recent studies of surgically resected patients with stage I NSCLC have reported that the prognosis of patients who have part-solid tumors is significantly better than that of patients with pure-solid tumors, even if the solid components of both tumors have the same diameter.^22^, ^23^, ^24^, ^25^, ^26^, ^27^^,^^33^ This phenomenon was also confirmed in our study (Table 3). In addition, we found that the iSUVmax was a prognostic factor irrespective of the GGO status among patients with lung adenocarcinoma.

Recently, perioperative treatment strategies using immune checkpoint inhibitors have joined the list of the standard of care in patients with NSCLC with clinical stage II and III diseases.^34^^,^^35^ However, in this study, we showed that some patients with clinical stage I diseases, such as those with clinical stage IB with high iSUVmax, had a worse prognosis (Figure 4). We hope that this image-based harmonization for FDG-PET/CT (if validated with prospective clinical trials) may provide a simple yet reliable way to identify high-risk patients with clinical stage I NSCLC who may benefit from future clinical trials of neoadjuvant and adjuvant therapies in this patient subgroup.

### Study Limitations

This study has some limitations. One is the retrospective design with a relatively small cohort of patients. Furthermore, the cohort was a heterogeneous population in terms of variable follow-up imaging. Because not all patients at the participating institutions with clinical stage I NSCLC underwent FDG-PET/CT imaging (at least during the study period), selection bias may exist. Prospective validation studies will be needed before more widespread use of this promising harmonization method can be recommended.

## Conclusions

Our results suggest that the novel image-based harmonization method, used in this study, was superior to mathematical-based harmonization methods, and among the FDG-PET/CT parameters, iSUVmax was the most important marker to predict malignant potential as well as RFS and OS after pulmonary resection in patients with clinical stage I NSCLC.

### Conflict of Interest Statement

Dr Hamada has received lecture fees from AstraZeneca, Chugai Pharmaceuticals, and Ono Pharmaceuticals outside of the submitted work. Dr Suda has received research funding from Rain Therapeutics and Boehringer Ingelheim, has received honoraria from Boehringer Ingelheim, Chugai Pharmaceuticals, and AstraZeneca, and has been on the advisory board of AstraZeneca outside of the submitted work. Dr Koga has received research funding from Boehringer Ingelheim. Dr Soh has received honorarium from Ethicon, Intuitive, and Covidien outside of the submitted work. Dr Daisaki has received research funding from Nihon Medi-Physics outside of the submitted work. Dr Mitsudomi has received research funding from Boehringer Ingelheim, AstraZeneca, Taiho, Ono Pharmaceuticals, Merck Sharp & Dohme, Eli Lilly, and Chugai Pharmaceuticals; has received lecture fees from AstraZeneca, Boehringer Ingelheim, Chugai Pharmaceuticals, Pfizer, Bristol-Myers Squibb, Eli Lilly, Merck Sharp & Dohme, Novartis, Merck Biopharma, Ono Pharmaceuticals, and Takeda Pharmaceuticals; and has been on the advisory board of AstraZeneca, Amgen, Janssen Pharma, Merck Sharp & Dohme, Novartis, and Puma Biotech outside of the submitted work. All other authors reported no conflicts of interest.

The *Journal* policy requires editors and reviewers to disclose conflicts of interest and to decline handling or reviewing manuscripts for which they may have a conflict of interest. The editors and reviewers of this article have no conflicts of interest.

## Supplementary Data

STROBE-checklist
