# Supplementary material for: Prognostic role of preoperative fluorine-18 fluorodeoxyglucose-positron emission tomography/computed tomography with an image-based harmonization technique: A multicenter retrospective study
Source: JTCVS Open. 2023 Feb 14;14:502–22. doi: 10.1016/j.xjon.2023.02.004 (PMC10328817; doi:10.1016/j.xjon.2023.02.004)
Supplement: STROBE-checklist [file mmc1.docx]

STROBE Statement—checklist of items that should be included in reports of observational studies

|  | Item No. | Recommendation | Page  No. | Relevant text from manuscript |
| --- | --- | --- | --- | --- |
| **Title and abstract** | 1 | (*a*) Indicate the study’s design with a commonly used term in the title or the abstract | 6 | Methods  We retrospectively examined 495 clinical stage I NSCLC patients who underwent FDG-PET/CT examinations prior to pulmonary resection between 2013 and 2014 at four institutions. Three different harmonization techniques were applied and an image-based harmonization, which showed the best-fit results, was used in the further analyses to evaluate prognostic roles of FDG-PET/CT parameters. |
|  |  | (*b*) Provide in the abstract an informative and balanced summary of what was done and what was found | 6 | Results  Cut-off values of image-based harmonized FDG-PET/CT parameters, iSUVmax (maximum standardized uptake value), iMTV (metabolic tumor volume), and iTLG (total lesion glycolysis), were determined using receiver operating characteristic curves that distinguish pathologic high invasiveness of tumors. Among these parameters, only the iSUVmax was an independent prognostic factor in recurrence-free and overall- survivals in univariate and in multivariate analyses. High iSUVmax value was associated with squamous histology or lung adenocarcinomas with higher pathologic grades. In subgroup analyses defined by ground-glass opacity (GGO) status and histology or by clinical stages, the prognostic impact of iSUVmax was always the highest compared with other FDG-PET/CT parameters. |
| Introduction | | | |  |
| Background/rationale | 2 | Explain the scientific background and rationale for the investigation being reported | 8-9 | In early-stage NSCLCs, harmonization of FDG-PET/CT data has been attempted using mathematical-based harmonization methods 8, 12 that use an equation generated using an anthropomorphic body phantom that conformed to National Electrical Manufacturers Association standards 13 to reduce inter- and intra-scanner variability. Nakayama et al. performed harmonization by adjusting the solid component sizes of the tumors because the deviation from the true SUV depends on the solid component size of the tumors 8. In contrast, Okada et al. reported a harmonization method that calibrated SUVs by dividing the actual SUV by the SUVmean measured in the phantom background 12. However, these mathematical-based methods are considered inaccurate. Nakayama’s method would be inadequate for smaller tumors, including those with ground-glass opacities (GGO) with a small solid component, and the simplified Okada’s method would be inadequate because the differences in SUVs between institutions are nonlinear. Therefore, a novel image-based methods are becoming mainstream of FDG-PET/CT harmonization in other types of malignancies 14-18. |
| Objectives | 3 | State specific objectives, including any prespecified hypotheses | 9 | In this study, we performed a multicenter retrospective study to evaluate the difference in SUVs between the above two mathematical-based and a novel image-based harmonization methods using phantom data. Because we confirmed that the image-based one was better than the mathematical-based methods, we performed further analyses to evaluate the validity and prognostic roles of the FDG-PET/CT data, which were harmonized using the novel image-based technique in surgically-resected NSCLC patients with clinical stage I disease. We also incorporated GGO status and a novel pathological classification, both of which have recently attracted attention as important prognostic factors for surgically-resected NSCLCs 1, 2, 19, 20. |
| Methods | | | |  |
| Study design | 4 | Present key elements of study design early in the paper | 9-10 | We retrospectively extracted patient information from clinical databases of Kindai University Hospital, Hyogo College of Medicine Hospital, National Cancer Center Hospital, and Nippon Medical School Hospital. Among patients who underwent surgical resection for clinical stage I NSCLC in accordance with the Union for International Cancer Control Tumor-Node-Metastasis 8th edition guidelines 21 between January 2013 and December 2014, we included 495 patients who underwent pretreatment FDG-PET/CT examinations prior to pulmonary resection. The exclusion criteria comprised any of the followings: 1) received preoperative treatment; 2) confirmed small cell lung cancer; 3) underwent exploratory thoracotomy, or biopsy alone, 4) positive surgical margins, and 5) history of lung cancer or synchronous lung cancer. We also excluded those who underwent FDG-PET/CT examination at another hospital. |
| Setting | 5 | Describe the setting, locations, and relevant dates, including periods of recruitment, exposure, follow-up, and data collection | 9-10 | Inclusion criteria  We retrospectively extracted patient information from clinical databases of Kindai University Hospital, Hyogo College of Medicine Hospital, National Cancer Center Hospital, and Nippon Medical School Hospital. Among patients who underwent surgical resection for clinical stage I NSCLC in accordance with the Union for International Cancer Control Tumor-Node-Metastasis 8th edition guidelines 21 between January 2013 and December 2014, we included 495 patients who underwent pretreatment FDG-PET/CT examinations prior to pulmonary resection. The exclusion criteria comprised any of the followings: 1) received preoperative treatment; 2) confirmed small cell lung cancer; 3) underwent exploratory thoracotomy, or biopsy alone, 4) positive surgical margins, and 5) history of lung cancer or synchronous lung cancer. We also excluded those who underwent FDG-PET/CT examination at another hospital.  The median follow-up period for all 495 patients was 67 months. |
| Participants | 6 | (*a*) *Cohort study*—Give the eligibility criteria, and the sources and methods of selection of participants. Describe methods of follow-up  *Case-control study*—Give the eligibility criteria, and the sources and methods of case ascertainment and control selection. Give the rationale for the choice of cases and controls  *Cross-sectional study*—Give the eligibility criteria, and the sources and methods of selection of participants | 9-10 | Inclusion criteria  We retrospectively extracted patient information from clinical databases of Kindai University Hospital, Hyogo College of Medicine Hospital, National Cancer Center Hospital, and Nippon Medical School Hospital. Among patients who underwent surgical resection for clinical stage I NSCLC in accordance with the Union for International Cancer Control Tumor-Node-Metastasis 8th edition guidelines 21 between January 2013 and December 2014, we included 495 patients who underwent pretreatment FDG-PET/CT examinations prior to pulmonary resection. The exclusion criteria comprised any of the followings: 1) received preoperative treatment; 2) confirmed small cell lung cancer; 3) underwent exploratory thoracotomy, or biopsy alone, 4) positive surgical margins, and 5) history of lung cancer or synchronous lung cancer. We also excluded those who underwent FDG-PET/CT examination at another hospital. |
|  |  | (*b*) *Cohort study*—For matched studies, give matching criteria and number of exposed and unexposed  *Case-control study*—For matched studies, give matching criteria and the number of controls per case | NA |  |
| Variables | 7 | Clearly define all outcomes, exposures, predictors, potential confounders, and effect modifiers. Give diagnostic criteria, if applicable | 11-12 | FDG-PET/CT parameters  SUVmax was defined as maximum SUV within the target volume of the primary tumor and was determined using the following formula: concentration of radioactivity in the volume of interest (MBq/mL) × total body weight (kg)/injected radioactivity (g/MBq). The SUVmean was calculated as the summed SUV in each voxel in the target volume divided by the number of voxels within the target volume of the primary tumor. MTV was measured automatically inside the primary tumor volume of interest, with the margin threshold set at 40% of SUVmax. TLG was calculated as SUVmean × MTV, considering both metabolic activity and tumor burden. iSUVmax, iMTV, and iTLG were defined as the SUVmax, MTV, and TLG values calculated using the image-based harmonized FDG-PET/CT method in individual patients. Receiver operating characteristic curves were used to identify optimal iSUVmax, iMTV, and iTLG cutoff values for predicting high pathologic invasiveness in all patients and in each subgroup (Supplementary Figure 1). |
| Data sources/ measurement | 8* | For each variable of interest, give sources of data and details of methods of assessment (measurement). Describe comparability of assessment methods if there is more than one group | 10-12 | TS-CT evaluation  For all patients, preoperative TS-CT images were independently reviewed by two investigators, and patients were classified into part-solid or solid groups based on the presence of a GGO component, as described in previous reports 22-27. CT images were evaluated on a monitor with a window level of 600–700 Hounsfield units and a window width of 1500–2000 Hounsfield units. Solid components were defined as areas of increased opacification that completely obscured the underlying vascular structures on TS-CT images. GGO components were defined as areas of increased hazy density that did not obscure the underlying vascular structures 28, 29.  FDG-PET/CT examination and harmonization techniques  The participating institutions used different FDG-PET/CT scanner systems, namely Biograph Duo (Siemens Healthcare, Erlangen, Germany), Discovery 600 (GE Healthcare, Waukesha, WI, USA), Gemini TF (Philips Medical Systems, Eindhoven, The Netherlands), and Gemini GXL (Philips Medical Systems). Prior to the examination, patients fasted for at least 5 hours, and blood glucose was measured immediately prior to injection of FDG at 3.0–4.0 MBq/kg of body weight. None of the patients had a blood glucose level greater than 200 mg/dL. Approximately 60 minutes after the injection, static emission images were obtained, during which the patients were allowed to breathe normally. The experienced physicians (K.K. and H.K.), who were board-certified in both diagnostic radiology and nuclear medicine and who were blinded to the other imaging results or clinical and histopathologic data, retrospectively reviewed all of the FDG-PET/CT images.  Regarding the techniques for FDG-PET/CT harmonization, three different harmonization methods were compared using mathematical-based (please see references. 8, 12 for further details) and image-based methods. Image-based harmonization was performed using RAVAT (Nihon Medi-Physics Co., Ltd., Tokyo, Japan), which is a commercially available software package that harmonizes SUVs obtained with different PET/CT systems in a range advocated by the Japanese Society of Nuclear Medicine, using phantom data 16, 30. Stand-alone RAVAT software can quantify PET images, and the software is typically used to adjust spatial resolution to harmonize PET images using a three-dimensional Gaussian filter.  FDG-PET/CT parameters  SUVmax was defined as maximum SUV within the target volume of the primary tumor and was determined using the following formula: concentration of radioactivity in the volume of interest (MBq/mL) × total body weight (kg)/injected radioactivity (g/MBq). The SUVmean was calculated as the summed SUV in each voxel in the target volume divided by the number of voxels within the target volume of the primary tumor. MTV was measured automatically inside the primary tumor volume of interest, with the margin threshold set at 40% of SUVmax. TLG was calculated as SUVmean × MTV, considering both metabolic activity and tumor burden. iSUVmax, iMTV, and iTLG were defined as the SUVmax, MTV, and TLG values calculated using the image-based harmonized FDG-PET/CT method in individual patients. Receiver operating characteristic curves were used to identify optimal iSUVmax, iMTV, and iTLG cutoff values for predicting high pathologic invasiveness in all patients and in each subgroup (Supplementary Figure 1).  Pathologic evaluation  Pathologic diagnoses were made by expert pathologists (T.Ka., T.Y. and S.H.) in accordance with the World Health Organization classification. Lung adenocarcinoma was classified as adenocarcinoma in situ, minimally invasive adenocarcinoma, and invasive adenocarcinoma, which was further divided into lepidic predominant, acinar predominant, papillary predominant, micropapillary predominant, solid predominant, or invasive mucinous adenocarcinoma 19. As previously reported, the predominant pattern was defined as the pattern with the largest percentage throughout the tissue sample. Invasive adenocarcinomas were further classified into three groups, as follows: grade 1, lepidic predominant; grade 2, acinar or papillary predominant; and grade 3, solid or micropapillary predominant, in accordance with the predominant pattern-based grading system 19. |
| Bias | 9 | Describe any efforts to address potential sources of bias | None |  |
| Study size | 10 | Explain how the study size was arrived at | 9-10 | We retrospectively extracted patient information from clinical databases of Kindai University Hospital, Hyogo College of Medicine Hospital, National Cancer Center Hospital, and Nippon Medical School Hospital. Among patients who underwent surgical resection for clinical stage I NSCLC in accordance with the Union for International Cancer Control Tumor-Node-Metastasis 8th edition guidelines 21 between January 2013 and December 2014, we included 495 patients who underwent pretreatment FDG-PET/CT examinations prior to pulmonary resection. The exclusion criteria comprised any of the followings: 1) received preoperative treatment; 2) confirmed small cell lung cancer; 3) underwent exploratory thoracotomy, or biopsy alone, 4) positive surgical margins, and 5) history of lung cancer or synchronous lung cancer. We also excluded those who underwent FDG-PET/CT examination at another hospital. |

Continued on next page

| Quantitative variables | 11 | Explain how quantitative variables were handled in the analyses. If applicable, describe which groupings were chosen and why | 13 | Receiver operating characteristic curves for iSUVmax, iMTV, and iTLG to predict lymphatic/vascular invasion, pleural invasion, and/or nodal involvement (high pathologic invasiveness) were generated to determine the cutoff values that yielded optimal sensitivity and specificity in accordance with a previously reported method 12. |
| --- | --- | --- | --- | --- |
| Statistical methods | 12 | (*a*) Describe all statistical methods, including those used to control for confounding | 13 | Statistical analyses  Statistical analyses were performed using JMP software, version 15.0 (SAS Institute Inc., Cary, NC, USA). Only simple statistical analyses were performed in this study, and following the recent guidelines 31, 32, these analyses were performed by well-educated and experienced researchers. Continuous variables were compared using the Mann–Whitney U test, whereas categorical variables were compared using the Chi-squared test. Receiver operating characteristic curves for iSUVmax, iMTV, and iTLG to predict lymphatic/vascular invasion, pleural invasion, and/or nodal involvement (high pathologic invasiveness) were generated to determine the cutoff values that yielded optimal sensitivity and specificity in accordance with a previously reported method 12. Recurrence-free survival (RFS) was defined as the interval from the day of surgery to the first event (relapse or death from any cause). For patients who did not experience disease recurrence, RFS was censored at the last visit. Overall survival (OS) was defined as the interval from the day of surgery to death from any cause. OS was censored at the last visit. RFS and OS were analyzed using the Kaplan–Meier method, and statistical differences in RFS or OS between groups were compared using the log-rank test. Univariate and multivariate Cox proportional hazard regression analyses were performed to assess the prognostic impact of the clinical parameters on RFS and OS. A p-value <0.05 was considered statistically significant. |
|  |  | (*b*) Describe any methods used to examine subgroups and interactions | 13 | Univariate and multivariate Cox proportional hazard regression analyses were performed to assess the prognostic impact of the clinical parameters on RFS and OS. A p-value <0.05 was considered statistically significant. |
|  |  | (*c*) Explain how missing data were addressed | 10 | We also excluded those who underwent FDG-PET/CT examination at another hospital. |
|  |  | (*d*) *Cohort study*—If applicable, explain how loss to follow-up was addressed  *Case-control study*—If applicable, explain how matching of cases and controls was addressed  *Cross-sectional study*—If applicable, describe analytical methods taking account of sampling strategy | NA |  |
|  |  | (*e*) Describe any sensitivity analyses | 15-16 | In the analyses of RFS and OS for the entire cohort, iSUVmax and iTLG values separated patients’ outcomes significantly (Figure 2). In the multivariate analysis, we found that iSUVmax (hazard ratio (HR): 3.02, p<0.001 for RFS and HR: 3.66, p=0.003 for OS), but not iTLG, was a significant prognostic factor (Table 2).  In the subgroup analysis focusing on pure solid lung adenocarcinomas (Figure 3), a high iSUVmax value was again the only significant predictive factor of both poor RFS (p<0.001) and OS (p=0.015). In multivariate analysis, iSUVmax was a significant prognostic factor for RFS (HR: 3.18, p=0.001) and OS (HR: 2.72, p=0.017) (Table 3). In the other subgroups, such as part-solid adenocarcinoma and non-adenocarcinoma groups, high iSUVmax was consistently a significant poor prognostic factor for both RFS and OS (Supplementary Figure 2 and 3). In further subgroup analysis, we observed that iSUVmax was a significant prognostic factor in patients with part-solid adenocarcinoma (C/T ratio > 0.5), while those with C/T ratio ≤ 0.5 usually had low iSUVmax and had excellent survival outcome (Supplementary Figure 4). These results suggest that iSUVmax is the most important prognostic factor to predict poor RFS and OS in clinical stage I NSCLC patients and in subgroups defined by GGO status (excluding those with C/T ratio ≤ 0.5) and/or histology. In addition, iSUVmax was a significant prognostic factor in patients with clinical stage IA and IB NSCLC. It is of note that clinical stage IB patients with high iSUVmax had the worst RFS (5-y RFS: high iSUVmax group, 39%; low iSUVmax group, 68%, p=0.008; 5-y OS: high iSUVmax group, 60%; low iSUVmax group, 94%, p=0.001) (Figure 4). Adjuvant chemotherapy, tegafur/uracil or platinum doublet (if pathological nodal involvement was found), was administered for these patients according to the Japanese guideline and patients’ general conditions. We observed that high iSUVmax was associated with poor prognosis in patients with clinical stage IB disease irrespective of the administration of adjuvant chemotherapy (Supplementary Figure 5). We also evaluated the ability of iSUVmax as a predictor of poor prognosis comparing with a conventional mathematical based SUVmax (mSUVmax) 12. Among 244 patients with low-risk group judged by the mSUVmax, there were 33 patients who were re-classified into high-risk group by iSUVmax. As shown in Supplementary Figure 6, these patients showed poorer RFS and OS compared with patients who were low-risk by both of the mSUVmax and iSUVmax. While there were only 3 patients who were iSUVmax low but mSUVmax high. |
| Results | | | | |
| Participants | 13* | (a) Report numbers of individuals at each stage of study—eg numbers potentially eligible, examined for eligibility, confirmed eligible, included in the study, completing follow-up, and analysed | 9-10 | Among patients who underwent surgical resection for clinical stage I NSCLC in accordance with the Union for International Cancer Control Tumor-Node-Metastasis 8th edition guidelines 21 between January 2013 and December 2014, we included 495 patients who underwent pretreatment FDG-PET/CT examinations prior to pulmonary resection. |
|  |  | (b) Give reasons for non-participation at each stage | 10 | The exclusion criteria comprised any of the followings: 1) received preoperative treatment; 2) confirmed small cell lung cancer; 3) underwent exploratory thoracotomy, or biopsy alone, 4) positive surgical margins, and 5) history of lung cancer or synchronous lung cancer. We also excluded those who underwent FDG-PET/CT examination at another hospital. |
|  |  | (c) Consider use of a flow diagram | None |  |
| Descriptive data | 14* | (a) Give characteristics of study participants (eg demographic, clinical, social) and information on exposures and potential confounders | 14 | Patient characteristics  The clinicopathological characteristics of the included patients are summarized in Table 1A. Among the 495 patients, 79 (16%), 203 (41%), 128 (26%), and 85 (17%) patients had clinical stage IA1, IA2, IA3, and IB disease, respectively, in accordance with the current tumor-node-metastasis classification (8th edition). In our cohort, 324 patients (65%) had pure solid tumors. Among the 495 patients, 421 patients (85%), 36 patients (7%), and 38 patients (8%) received lobectomy, segmentectomy and wedge resection, and 383 patients (77%) underwent mediastinal lymph node dissection. Lymphatic invasion, vascular invasion, pleural invasion, and pathologic nodal involvement (pN1 or pN2) was recorded in 105 (21%), 172 (35%), 99 (20%), and 59 (12%) patients, respectively. |
|  |  | (b) Indicate number of participants with missing data for each variable of interest | NA |  |
|  |  | (c) *Cohort study*—Summarise follow-up time (eg, average and total amount) | 10 | The median follow-up period for all 495 patients was 67 months. |
| Outcome data | 15* | *Cohort study*—Report numbers of outcome events or summary measures over time | NA |  |
|  |  | *Case-control study—*Report numbers in each exposure category, or summary measures of exposure | 15 | We also analyzed the correlations between the FDG-PET/CT parameters and the histological findings. As shown in Table 1B, most of squamous cell carcinoma patients (94%) were classified into the high iSUVmax group, while half of the lung adenocarcinoma patients were classified into this group. Among lung adenocarcinomas, the percentages of patients who were classified into the high iSUVmax group (≥ 2.3) were 14%, 57%, and 83% for predominant pattern grade 1, grade 2, and grade 3 tumors, respectively. Such hierarchy was not evident in iMTV (66%, 79%, and 73%, for predominant pattern grade 1, grade 2, and grade 3, respectively) or in iTLG (28%, 53%, and 63%, respectively). These results suggest that a high iSUVmax value was the most important predictor of lung adenocarcinomas with higher pathologic grade. |
|  |  | *Cross-sectional study—*Report numbers of outcome events or summary measures | NA |  |
| Main results | 16 | (*a*) Give unadjusted estimates and, if applicable, confounder-adjusted estimates and their precision (eg, 95% confidence interval). Make clear which confounders were adjusted for and why they were included | 15-16 | In the analyses of RFS and OS for the entire cohort, iSUVmax and iTLG values separated patients’ outcomes significantly (Figure 2). |
|  |  | (*b*) Report category boundaries when continuous variables were categorized | 14 | Cut-off values for iSUVmax, iMTV, and iTLG was determined using ROC curves that distinguish tumors with high pathologic invasiveness (Supplementary Figure S1). Among three FDG-PET/CT parameters, iSUVmax had the highest sensitivity and specificity (area under the curve: AUC = 0.811) in all patients compared with iMTV (AUC = 0.562) and iTLG (AUC = 0.740). |
|  |  | (*c*) If relevant, consider translating estimates of relative risk into absolute risk for a meaningful time period | 15 | In the multivariate analysis, we found that iSUVmax (hazard ratio (HR): 3.02, p<0.001 for RFS and HR: 3.66, p=0.003 for OS), but not iTLG, was a significant prognostic factor (Table 2). |

Continued on next page

| Other analyses | 17 | Report other analyses done—eg analyses of subgroups and interactions, and sensitivity analyses | 15-16 | In the subgroup analysis focusing on pure solid lung adenocarcinomas (Figure 3), a high iSUVmax value was again the only significant predictive factor of both poor RFS (p<0.001) and OS (p=0.015). In multivariate analysis, iSUVmax was a significant prognostic factor for RFS (HR: 3.18, p=0.001) and OS (HR: 2.72, p=0.017) (Table 3). In the other subgroups, such as part-solid adenocarcinoma and non-adenocarcinoma groups, high iSUVmax was consistently a significant poor prognostic factor for both RFS and OS (Supplementary Figure 2 and 3). In further subgroup analysis, we observed that iSUVmax was a significant prognostic factor in patients with part-solid adenocarcinoma (C/T ratio > 0.5), while those with C/T ratio ≤ 0.5 usually had low iSUVmax and had excellent survival outcome (Supplementary Figure 4). These results suggest that iSUVmax is the most important prognostic factor to predict poor RFS and OS in clinical stage I NSCLC patients and in subgroups defined by GGO status (excluding those with C/T ratio ≤ 0.5) and/or histology. In addition, iSUVmax was a significant prognostic factor in patients with clinical stage IA and IB NSCLC. It is of note that clinical stage IB patients with high iSUVmax had the worst RFS (5-y RFS: high iSUVmax group, 39%; low iSUVmax group, 68%, p=0.008; 5-y OS: high iSUVmax group, 60%; low iSUVmax group, 94%, p=0.001) (Figure 4). Adjuvant chemotherapy, tegafur/uracil or platinum doublet (if pathological nodal involvement was found), was administered for these patients according to the Japanese guideline and patients’ general conditions. We observed that high iSUVmax was associated with poor prognosis in patients with clinical stage IB disease irrespective of the administration of adjuvant chemotherapy (Supplementary Figure 5). We also evaluated the ability of iSUVmax as a predictor of poor prognosis comparing with a conventional mathematical based SUVmax (mSUVmax) 12. Among 244 patients with low-risk group judged by the mSUVmax, there were 33 patients who were re-classified into high-risk group by iSUVmax. As shown in Supplementary Figure 6, these patients showed poorer RFS and OS compared with patients who were low-risk by both of the mSUVmax and iSUVmax. While there were only 3 patients who were iSUVmax low but mSUVmax high. |
| --- | --- | --- | --- | --- |
| Discussion | | | | |
| Key results | 18 | Summarise key results with reference to study objectives | 17 | To the best of our knowledge, this is the first clinical study that used a novel image-based harmonization method for FDG-PET/CT parameters to evaluate prognostic factors in patients who received pulmonary resection for clinical stage I NSCLCs in multi-institutions (with different FDG-PET/CT machines). We observed that the novel image-based harmonization method was superior to previously reported mathematical methods, in addition, we observed that iSUVmax was the most important FDG-PET/CT parameter in terms of prognostic markers for RFS and OS as well as the predictor of histological grades among lung adenocarcinoma patients. Lastly, we found that iSUVmax can identify patients with poor prognosis among those with low-risk group judged by a mathematical method. |
| Limitations | 19 | Discuss limitations of the study, taking into account sources of potential bias or imprecision. Discuss both direction and magnitude of any potential bias | 18 | This study has some limitations. One is the retrospective design with a relatively small cohort of patients. Furthermore, the cohort was a heterogeneous population in terms of variable follow-up imaging. Because not all patients at the participating institutions with clinical stage I NSCLC underwent FDG-PET/CT imaging (at least during the study period), selection bias may exist. |
| Interpretation | 20 | Give a cautious overall interpretation of results considering objectives, limitations, multiplicity of analyses, results from similar studies, and other relevant evidence | 18 | Our results suggest that the novel image-based harmonization method, used in this study, was superior to mathematical-based harmonization methods, and among the FDG-PET/CT parameters, iSUVmax was the most important marker to predict malignant potential as well as RFS and OS after pulmonary resection in clinical stage I NSCLC patients. |
| Generalisability | 21 | Discuss the generalisability (external validity) of the study results | 17 | The importance of SUVmax, as a prognostic marker in clinical stage I diseases, was reported in a previous study, although the main results of the study were the usefulness of MTV and TLG in the total cohort (clinical stage I–II NSCLCs) in multivariate analysis 6. Therefore, we consider that our result is consistent with the previous one, since our cohort enrolled stage I disease only. In small-sized NSCLCs, it is hypothesized that the simple SUVmax, rather than the factors that include volume elements, would be more useful as a predictor of pathologic invasiveness, pathologic grade, and prognosis. Furthermore, it is of note that subgroup analysis in our study, based on histology and the GGO status, showed that iSUVmax was consistently better than iMTV and iTLG to predict RFS and OS. Recent studies of surgically-resected stage I NSCLC patients have reported that the prognosis of patients who have part-solid tumors is significantly better than that of patients with pure solid tumors, even if the solid components of both tumors have the same diameter 22-27, 33. This phenomenon was also confirmed in our study (Table 2), in addition, we found that the iSUVmax was also a prognostic factor irrespective of the GGO status among lung adenocarcinoma patients. |
| Other information | |  | | |
| Funding | 22 | Give the source of funding and the role of the funders for the present study and, if applicable, for the original study on which the present article is based | 3 | This study was supported by grants-in-aid for scientific research from the Japan Society for the Promotion of Science (grant 20K17763 to Dr Hamada, grant 19K08187 to Dr Kitajima, grant 22K07291 to Dr Suda, grant 22K08986 to Dr Soh, and grant 20H03773 to Dr Mitsudomi). |

*Give information separately for cases and controls in case-control studies and, if applicable, for exposed and unexposed groups in cohort and cross-sectional studies.

**Note:** An Explanation and Elaboration article discusses each checklist item and gives methodological background and published examples of transparent reporting. The STROBE checklist is best used in conjunction with this article (freely available on the Web sites of PLoS Medicine at http://www.plosmedicine.org/, Annals of Internal Medicine at http://www.annals.org/, and Epidemiology at http://www.epidem.com/). Information on the STROBE Initiative is available at www.strobe-statement.org.
